# The Role of Carrageenan and Carboxymethylcellulose in the Development of Intestinal Inflammation

**DOI:** 10.3389/fped.2017.00096

**Published:** 2017-05-01

**Authors:** John Vincent Martino, Johan Van Limbergen, Leah E. Cahill

**Affiliations:** ^1^Pediatric Gastroenterology, Hepatology and Nutrition, IWK Health Centre, Halifax, NS, Canada; ^2^Medicine, Dalhousie University, Halifax, NS, Canada; ^3^Nutrition, Harvard T.H. Chan School of Public Health, Boston, MA, USA

**Keywords:** carboxymethylcellulose, carrageenan, inflammatory bowel disease, microbiota, Crohn’s disease, ulcerative colitis

## Abstract

Although the exact pathophysiology remains unknown, the development of inflammatory bowel disease (IBD) is influenced by the interplay between genetics, the immune system, and environmental factors such as diet. The commonly used food additives, carrageenan and carboxymethylcellulose (CMC), are used to develop intestinal inflammation in animal models. These food additives are excluded from current dietary approaches to induce disease remission in Crohn’s disease such as exclusive enteral nutrition (EEN) using a polymeric formula. By reviewing the existing scientific literature, this review aims to discuss the role that carrageenan and CMC may play in the development of IBD. Animal studies consistently report that carrageenan and CMC induce histopathological features that are typical of IBD while altering the microbiome, disrupting the intestinal epithelial barrier, inhibiting proteins that provide protection against microorganisms, and stimulating the elaboration of pro-inflammatory cytokines. Similar trials directly assessing the influence of carrageenan and CMC in humans are of course unethical to conduct, but recent studies of human epithelial cells and the human microbiome support the findings from animal studies. Carrageenan and CMC may trigger or magnify an inflammatory response in the human intestine but are unlikely to be identified as the sole environmental factor involved in the development of IBD or in disease recurrence after treatment. However, the widespread use of carrageenan and CMC in foods consumed by the pediatric population in a “Western” diet is on the rise alongside a corresponding increase in IBD incidence, and questions are being raised about the safety of frequent usage of these food additives. Therefore, further research is warranted to elucidate the role of carrageenan and CMC in intestinal inflammation, which may help identify novel nutritional strategies that hinder the development of the disease or prevent disease relapse post-EEN treatment.

## Introduction

Crohn’s disease (CD) is a chronic relapsing and remitting inflammatory bowel disease (IBD) that causes damage to the mucosal lining of the gastrointestinal tract, resulting in abdominal pain, (bloody) diarrhea, intestinal ulceration, and often progression toward stricturing/penetrating complications requiring surgery, malnutrition, impaired growth, disability, and even mortality (1, 2). Epidemiological studies have shown an increasing incidence of IBD [composed of CD and ulcerative colitis (UC)] both in areas of the world where the disease has been originally more prevalent (North America, Australia, and Europe) and in countries where IBD was previously infrequent (Asia and South America) (3, 4). It is noteworthy that this increase has occurred in conjunction with the transition toward a “Western” diet characteristically high in processed foods and fats and low in fruits and vegetables (5). Although the exact etiology and pathogenesis of IBD remain obscure, it is believed to result from a combination of genetic, microbial, immunological, and environmental factors including diet (6). In the last couple of years, the field of nutrition in IBD has advanced substantially (7, 8), with several dietary aspects of interest including the food additives used to induce intestinal inflammation and ulceration in animal models of IBD (9–11).

Food additives are substances intentionally added during production, processing, packaging, transportation, or storage of commercial food products. Carrageenan is the name referring to a family of high-molecular-weight sulfated polysaccharides extracted from seaweeds and commonly used as a thickening and emulsifying food additives to improve the texture of commercial food products including infant formulas, dairy products, milk alternatives such as almond milk, processed meats, and soy-based products (9, 12). First patented in the United States in the 1930s, carrageenan’s use as a food additive in the Western diet has been substantially increasing over the last 50 years (9, 13). Carboxymethylcellulose (CMC) is a derivative of cellulose, making it affordable and abundant with thickening and emulsifying qualities, and it is found vastly throughout the commercial food industry with a progressive annual increase in its usage as a food additive (14, 15).

## The Role of Carrageenan in Intestinal Inflammation in Animal Models

It has been demonstrated that when guinea pigs are supplied with degraded carrageenan in their drinking water, ulcerations develop in 100% of the animals in their large intestine by the end of a 30-day period (16). The lesions induced by carrageenan in the guinea pigs’ large bowel resemble features of human UC (10). Carrageenan has also produced ulcerative lesions in rabbits, mice, and rats that were associated with weight loss, anemia, diarrhea, visible or occult blood, and sometimes mucus in the feces (17). Further, macroscopic features reminiscent of human UC, such as ulcerations predominantly limited to the mucosa along with pseudopolyps and polypoidal formations, were observed, as well as features commonly seen in CD such as strictures in the small intestine leading to obstruction (17). Histological changes associated with exposure to carrageenan in animal models include acute, subacute, and chronic inflammatory changes in the mucosa; occasional crypt abscesses; cystic dilatation or distortion of mucosal glands; mucosal ulceration in various stages of progression and healing; and hyperplastic changes of the glandular epithelium (17). Pricolo et al. noted that animals fed a 2% solution of carrageenan mixed with standard rat chow developed small bowel lesions first (from 2 to 6 weeks) followed by colonic lesions that developed after 8 weeks (11), which may be of particular interest for a subgroup of human CD patients who extend their disease location (18–20).

In recent years, evidence from animal studies has been mounting to suggest that the gut microbiome plays a critical role in the development of inflammation in response to exposure to carrageenan. For example, guinea pigs fed degraded carrageenan develop cecal ulcerations within 21–30 days after carrageenan treatment (21), whereas germ-free guinea pigs fed carrageenan for 6 months or more do not develop any intestinal lesions (22). Concurrent treatment of conventional guinea pigs with carrageenan and metronidazole, an antimicrobial primarily active against anaerobic bacteria, has been shown to prevent the development of intestinal ulcerations (22, 23). Consistent with recent reports in human IBD (24, 25), microorganisms such as *Bacteroide*s *vulgatus* appear to play an important role in the development of the experimental ulcerative lesions in animals (26). For example, animals who were immunized with *B. vulgatus*, prior to carrageenan administration developed experimental disease at a faster rate and more severe lesions than animals that were administered carrageenan alone (23, 27). Mirroring the development of antibodies to components of the intestinal microbiome in human IBD (28, 29), Onderdonk et al. noted that non-immune carrageenan recipients developed antibodies to *B. vulgatus* during carrageenan treatment, implying that an adaptive immune response occurs in the animals following carrageenan-induced intestinal ulceration (26, 27). Factors present on the bacterial outer membrane may mediate these antibody responses, as one study assessed eight strains of *B. vulgatus* and observed the alpha phenon (term used to reflect strain variation of outer membrane proteins) was associated with the enhancement of colitis (30).

The specific mechanism by which carrageenan induces inflammation in experimental animal models is not clearly defined, but carrageenan has been demonstrated to decrease the amount of epithelial glycoproteins in the colon (31) and is capable of inhibiting the interaction between macrophages and lymphocytes (32). The involvement of toll-like receptor-4 (TLR4) and interleukin (IL)-6 in the innate immune response to carrageenan was demonstrated using TLR4- and myeloid differentiation primary response 88-deficient mice (33). Recently, Wei et al. showed in a trinitrobenzenesulfonic acid (TNBS) model of colitis in BALB/c mice that pretreatment with carrageenan aggravated the severity (both clinical and histological) of TNBS colitis, with a concomitant increased expression of IL-6 and tumor necrosis factor alpha (TNF)-α and a reduction in IL-10 (34). Taken together, these animal studies have led to the hypothesis that food emulsifiers such as carrageenan may act as a conditional inflammatory agent that magnifies any existing chronic inflammation of the intestinal tract provoked by pathogens (35). This hypothesis explains why carrageenan has been found to induce intestinal inflammation in most animal studies, but not all. For example, healthy neonatal pigs fed infant formula with carrageenan for 28 days had no effect on blood cytokine evaluations (IL-1β, IL-6, IL-8, and TNF-α) (36), but they would not have had any baseline inflammation and were not exposed to pathogens.

## The Role of CMC in Intestinal Inflammation in Animal Models

In mice given relatively low concentrations of CMC, low-grade inflammation and obesity/metabolic syndrome was induced in wild-type hosts and promoted robust colitis in IL-10^−/−^ and TLR5^−/−^ mice. In the control mice not fed CMC, the closest bacteria were shown to reside about 25 µm from epithelial cells with no bacteria observed within 10 µm, whereas CMC-treated mice exhibited some bacteria in apparent contact with the epithelium, and the average distance was reduced by more than 50% (37). In this study, CMC dramatically altered microbiota composition in both fecal and intestinal-adherent bacteria, and the authors concluded that chronic exposure to CMC resulted in erosion of the protective function of the mucus, increased bacterial adherence, and a more pro-inflammatory microbiota (37). More recently, Chassaing et al. tried to disentangle the effect of inflammation itself on microbiota composition from the effect of CMC on host parameters (that in turn promotes inflammation and subsequently alters the microbiota). They used the Mucosal Simulator of the Gastrointestinal Microbial Ecosystem (M-SHIME) model to examine the effects of emulsifiers on the human microbiota *in vitro* (38). CMC directly acted upon human microbiota to increase its pro-inflammatory potential; the CMC-induced increase in flagellin occurred after 1 day and was driven by altered microbiota gene expression rather than microbiota composition changes (38). Transfer of both emulsifier-treated M-SHIME microbiotas to germ-free recipient mice recapitulated many of the host and microbial alterations observed in mice directly treated with emulsifiers, notably the development of intestinal inflammation (38). These results suggest that the microbiota may be a key direct target of CMC to drive chronic intestinal inflammation.

Swidsinski et al. have demonstrated that exposure to 2% CMC in IBD-susceptible IL-10 gene-deficient mice results in “CD-like effects,” reporting that changes in the CMC-treated mice were identical to ileal biopsy findings of CD patients (14). This was supported by the observations that mice treated with 2% CMC demonstrated increased concentrations of bacteria in the ileum, larger spaces between villi, increased amounts of bacteria adherent to the villi, increased white blood cells in the lumen, and, in some of the mice, bacterial infiltration of the epithelium (14).

These findings of the effects of commonly used emulsifiers on the microbiota, mucus layer, and epithelial barrier integrity should be considered in conjunction with the recent work by Desai et al. (39) regarding the role of dietary fiber on mucus barrier maintenance. These authors demonstrated that in gnotobiotic mice colonized with a synthetic human gut microbiota and denied dietary fiber, the microbiota will consume host-secreted mucus glycoproteins as a nutrient source in place of fiber, leading to erosion of the colonic mucus barrier (39). In turn, this erosion of the colonic mucous barrier promoted greater epithelial access and lethal colitis by the murine pathogen *Citrobacter rodentium* (39). Carrageenan and CMC are often added to commercial food products as a fiber (40), instead of other dietary fiber (such as water-insoluble cellulose and resistant starch or water-soluble fiber such as pectin and raffinose) sources. In addition, many CD patients already limit dietary fiber to avoid its bulk-forming and laxative effects. Therefore, a “perfect storm” setting in which CD patients increase their CMC/carrageenan intake but limit their intake of other dietary fibers is created, which could propagate or exacerbate the existing dysbiosis toward more mucin-degradation and which could enhance the susceptibility for mucosa-associated pathogens.

## Food Additives and Intestinal Inflammation in Humans

**Figure 1:**
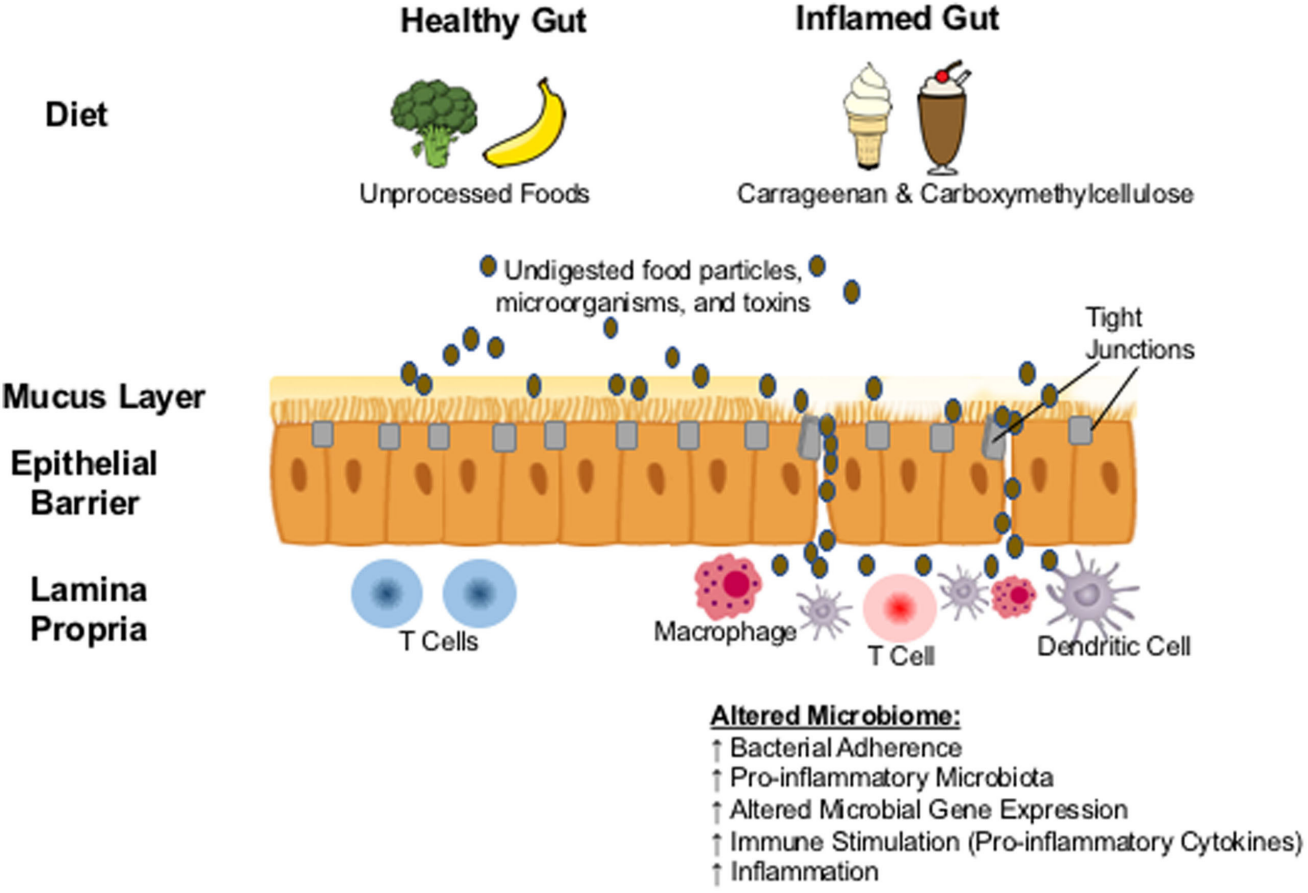
**Proposed biological mechanism: carrageenan and carboxymethylcellulose in processed foods result in the erosion of the protective mucus layer and in the abnormal expression of tight junction proteins**. Undigested food particles, toxins, and microorganisms are then able to pass though the intestinal epithelium resulting in translocation of microbes and the overcolonization by pathobionts that can alter the composition of the gut microbiome, triggering the activation of the immune system and the development of inflammation.

The reports above of commonly used food additives causing intestinal inflammation in animal models have triggered investigations of how they may lead to inflammation in the human gastrointestinal tract, although perfectly corresponding human studies are of course unethical to conduct. It is likely that mechanisms by which carrageenan induces inflammation in the human intestine are similar to animals and multifactorial: the stimulation of pro-inflammatory cytokines, the disruption of the epithelial barrier, and the interference with innate mucosal immune responses to microorganisms (Figure 1). Community-level metabolic networks within the microbiome produce bioactive metabolites that have an established role in intestinal immune homeostasis and a healthy gut mucosal barrier (41, 42). This barrier protecting the host from luminal antigens is composed of enterocytes (epithelial cells) and the seal between them provided by the tight junctions (43). With a healthy gut mucosal barrier, an individual maintains a homeostasis where the passage of nutrients, ions, and water across the intestinal epithelium into the mesenteric blood stream is tightly regulated, while translocation of dietary antigens and components of the microbiome is prevented (43), and the active crosstalk between a diverse microbiome and the intestinal immune system leads to immune tolerance. Impaired barrier function can lead to abnormalities in the expression of tight junction protein that may trigger immune activation and the development of inflammatory disease in susceptible individuals (43, 44).

Borthakur et al. provided the first report of the inflammatory response of human intestinal epithelial cells to carrageenan exposure by demonstrating that carrageenan stimulates an inflam-atory cascade in colonic epithelial cells in a pathway involving B-cell lymphoma/leukemia 10 gene (Bcl10) activation and the increased production of IL-8 (9), a key pro-inflammatory cytokine. These results were supported when Choi et al., using the human colonic adenocarcinoma cell line HCT-8, demonstrated that carrageenan led to the activation of nuclear factor kappaB, which subsequently increased the gene induction of IL-8 (12). In addition, they reported that carrageenan triggered a disruption of the epithelial barrier, decreasing the density of tight junctional protein zonula occludens (ZO)-1, causing disarray in the distribution of ZO-1 throughout the epithelium, and decreasing the gene expression of this tight junction protein (12). More recently, this group showed that carrageenan exposure triggered the expression of the proapoptotic macrophage inhibitory cytokine 1 (MIC1), which is in turn counteracted by MIC1-induced expression of activating transcription factor 3 (45).

Carrageenan has been reported to interact with the glycoprotein deleted in malignant brain tumors 1 (DMBT1). DMBT1 functions as a pattern recognition molecule with a peptide domain capable of binding and aggregating a wide spectrum of bacteria (46) and has been demonstrated to prevent the invasion of bacteria into intestinal epithelial cells *in vitro* (47). Carrageenan inhibits the bacterial aggregating function of DMBT1 *via* binding to the specific peptide that recognizes bacteria, and it has been suggested that carrageenan disrupts the mucosal protection provided by DMBT1 (48). This raises the possibility that carrageenan is capable of disrupting an innate mucosal immune function provided by DMBT1, which can then trigger the initiation or perpetuation of an inflammatory response to intestinal bacteria or bacterial antigens (48).

In human cells, carrageenan has also been demonstrated to trigger innate immune responses through pathways that involve Bc110, TLR4, NF-κB, and AP-1 (9, 35, 49), leading to the upregulation of TNF-α secretion (50). For example, Jiang et al. (51) demonstrated that carrageenan-induced TNF-α secretion is the main contributor to cellular damage in Caco-2 monolayers exposed to carrageenan. However, researchers in the field note that in these studies, the degree of inflammation caused by carrageenan alone is low, while the inflammation generated through these pathways when a pathogen is additionally present is high, supporting the hypothesis that an interaction effect is present in which carrageenan serves as a pro-inflammatory agent to amplify existing intestinal inflammation (35).

In view of the recent benchmark reports by Chassaing et al. (38) and Desai et al. (39), more studies of the intestinal response to the widely used emulsifiers, carrageenan and CMC, together with other features of a Western diet such as reduced dietary fiber intake are eagerly awaited (52). It is noteworthy that the Food and Drug Administration regulations do not contain a definition of dietary fiber but rather have relied on analytical methods for measuring levels of dietary fiber present in food. Therefore, an isolated or synthetic non-digestible carbohydrate such as carrageenan and CMC can be added to foods and quantified as a dietary fiber even if it does not provide the beneficial physiological effect to human health that dietary fiber should provide. For example, CMC is listed among the 26 most common fibers being added to food and declared on the Nutrition Facts label as dietary fiber (40).

## Widespread Use of Food Additives

Patented in the United States in the 1930s, carrageenan was granted GRAS (generally regarded as safe) status in 1959 and currently remains included as a food additive that holds GRAS status in the Code of Federal Regulations in the United States (13). Carrageenan acts to thicken, stabilize, and emulsify a wide variety of foods typically consumed in the Western diet including dairy products such as chocolate milk, ice cream, cottage cheese, sour cream, and yogurt; processed meats; soymilk; almond milk; mayonnaise; and infant formula (53). Estimates regarding the average daily intake of carrageenan vary from 20 to 200 mg/day (13, 54) and are currently difficult to put into context without a standard for comparison within human studies or with animal studies.

Carboxymethylcellulose is used broadly throughout the food industry in products typically consumed by children including candy, chewing gum, “snack foods,” ketchup, and various baked goods, and currently, there are no quantitative restrictions on its use nor does its addition to food require to be declared (14, 15). CMC is listed in the Food and Drug Administration’s database of GRAS substances (55), and CMC is also included in the Safety and Toxicity of Excipients for Paediatrics (STEP) database (56, 57), a resource developed by the European Paediatric Formulation Initiative and the United States Paediatric Formulation Initiative in collaboration for storage and rapid/effortless access to the safety and toxicological data of commonly used excipients.

## Role of Diet in IBD: Epidemiology and Treatment

The increasing incidence rate of IBD worldwide, with the highest incidence and prevalence being in Westernized countries (3), has been associated with increased consumption of a Western diet (5). A case–control study conducted in Japan assessed environmental risk factors for UC in 101 patients and observed that consumption of foods typical of a Western diet including bread, butter, and sausage was significantly related to an increase in UC risk (58). Moreover, D’Souza et al. demonstrated that the consumption of fried and fast foods, meats, snacks, and desserts, which they labeled as a traditional Western dietary pattern, increased the likelihood of acquiring CD in adolescent females (59). Conversely, they found that both males and females exposed to a prudent dietary pattern, consisting of mostly vegetables, fish, grains, and nuts, had a decreased likelihood of developing CD (59).

Exclusive enteral nutrition (EEN) is recommended as a first-line therapy in pediatric CD and involves the use of a liquid formula diet as the only source of nutrition over several weeks (8, 60).The mechanism of action of EEN has not been clearly established; however, it may provide efficacy *via* eliminating certain components of the Western diet and through modulation of the microbiome (61–67). More recently, novel dietary therapy regimens have been described, which involve avoidance of processed foods such as the CD exclusion diet (CDED) (68) and the Specific Carbohydrate Diet (69). Sigall-Boneh et al. demonstrated that the CDED, which avoids exposure to many components of a Western diet such as dairy products and processed foods containing food additives, led to remission in 70% of patients (8). Through large network studies such as the Canadian Institutes for Health Research supported Inflammation, Microbiome, Alimentation, GastroIntestinal and Neuropsychiatric effects (IMAGINe), considerable efforts are being made to investigate the potential contributions of dietary factors to IBD pathophysiology as a dysregulated interplay of genetic, environmental, microbial, and immunological factors (6, 59), and so more details on the role of diet, including food additives such as carrageenan and CMC, in IBD may be discovered in the near future and inform dietary recommendations and medical treatment of IBD.

## Conclusion

Carrageenan and CMC administered in animal models consistently result in intestinal ulcerations with histopathological features similar to human IBD. Although the set of precise mechanisms through which these emulsifiers induce lesions and inflammation remains unknown, disruption of the epithelial barrier and dysregulation of the immune response to the gut microbiome have been repeatedly implicated. These findings raise concern because carrageenan and CMC are used extensively in processed food products that are consumed by the pediatric population, and the incidence rate of childhood IBD is increasing concurrently with a rise in the adoption of a Western diet. The only successful dietary interventions to have induced CD remission exclude processed foods containing carrageenan and CMC, further supporting the possibility that carrageenan and CMC are potential triggering or magnifying substances of inflammation in IBD. Further research is warranted to clarify the role of carrageenan and CMC in the microbiome alteration of intestinal inflammation together with an improved appreciation of the complex interplay with the consumption of dietary fibers, and such studies could lead to novel nutritional strategies that help prevent the development of IBD or help induce and sustain remission.

## Author Contributions

All authors listed have made substantial, direct, and intellectual contribution to the work and approved it for publication.

## Conflict of Interest Statement

JVL has participated in an advisory board for Nestlé and has received speaking fees and research support from Nestlé. The other authors declare no conflict of interest.
